# Food groups intake in relation to stunting among exceptional children

**DOI:** 10.1186/s12887-020-02291-7

**Published:** 2020-08-20

**Authors:** Seyyed Mostafa Nachvak, Omid Sadeghi, Shima Moradi, Ahmad Esmailzadeh, Roghayeh Mostafai

**Affiliations:** 1 Department of Nutritional Sciences, Research Center for Environmental Determinants of Health (RCEDH), Health Institute, Kermanshah University of Medical Sciences, Kermanshah, Iran; 2 Department of Community Nutrition, School of Nutritional Sciences and Dietetics, Tehran University of Medical Sciences, Tehran, Iran; 3 Department of Nutrition & Biochemistry, School of Public Health, Tehran University of Medical Sciences, Tehran, Iran

**Keywords:** Diet, Exceptional children, Food, Height, Stunting

## Abstract

**Background:**

Although several studies have examined the link between different food groups intake and stunting among children, no study, to our knowledge, was done on exceptional children. The aim of this study was assessed the association of dietary intake and stunting in Iranian exceptional children.

**Methods:**

This cross-sectional study was conducted on 470 exceptional children (226 mentally retarded, 182 deaf and 62 blind children), aged 5–15 years. Height was measured using standard tool. Stunting was defined as height-for-age z-score of <-1. A validated dietary habit questionnaire was applied to assess dietary intakes.

**Results:**

Mean age of children was 10.02 ± 2.04 years. Stunting was prevalent among 50.6% of children. Compared with children in the lowest category of dairy consumption, those in the highest category had lower odds of stunting. This association remained significant even after adjusting for covariates (OR: 0.50, 95% CI: 0.29–0.87). In addition, moderate consumption of egg (1–3 time/wk) was inversely associated with stunting either before or after controlling for potential confounders (OR: 0.36, 95% CI: 0.21–0.64). Such finding was also seen among mentally retarded children (OR: 0.38, 95% CI: 0.16–0.89). No other significant association was seen between intakes of other food groups (including meat, fruits and vegetables) and stunting.

**Conclusions:**

We found that higher intake of dairy products and egg was associated with lower risk of stunting. However, intakes of other food groups including meat, fruits and vegetables were not significantly related to stunting.

## Background

Stunting is a major public health problem in developed and developing countries [1, 2]. Based on world health organization (WHO), stunting is defined as gender-specific height-for-age value of less than two standard deviations of the WHO Child Growth Standards [3]. Approximately, 215 million children, around the world, are stunted. National estimates in Iran showed that 4.7% of Iranian children were affected [4]. Children with stunting might suffer from current, and possibly later, delayed mental and motor development as well as limited work-capacity due to reduced muscle mass [4, 5]. Therefore, finding appropriate strategies to prevent stunting is of great importance.

It has been shown that environmental factors, in particular diet, have an important role in stunting [5]. It can be developed from inadequate intake of food, inappropriate quality of diet, or a combination of both [6]. A large number of studies were published on growth-limiting nutrients indicating primary deficiency of zinc, vitamin A, vitamin D and iron, along with insufficient intake of protein and energy [7–9]. However, less attention has been laid down on the consumption of food groups including dairy, meat, vegetables and fruits in relation to stunting in children. These food groups contain a high amount of fibers, antioxidants and essential minerals including magnesium, calcium and iron [10, 6]. These micronutrients might be involved in height growth [6]. Therefore, assessing the association between consumption of these food groups and height growth might present new strategies to prevent stunting.

To the best of our knowledge, no study has assessed the association of some food groups intake including fruits, vegetable, dairy and meat with stunting in exceptional children. However, among adults, it has been shown that compared with those with the lowest adherence, women with greater adherence to the prudent dietary pattern in childhood had higher height [11]. Assessing food intake in relation to stunting is more important in exceptional children because they might be mentally retarded, blind and deaf. Given the high prevalence of stunting in Iranian children, current study aimed to assess the association of fruits, vegetables, dairy and meat consumption with stunting in Iranian exceptional children.

## Methods

### Participants

This cross-sectional study was conducted on exceptional children aged between 5 and 15 years in 2014. In Iran, children at the age of six are assessed for cognitive, visual and auditory tests by standardized tests in places administered by the Ministry of Health and the Ministry of Education. If the child does not have any problem, he / she will be referred to normal schools for elementary education. However, if there are any cognitive, visual or hearing problems, they are referred to special schools that have been set up to educate children with special needs. The method of teaching in these schools is different from normal schools. Exceptional school teachers undergo a series of special trainings and courses to educate exceptional children and then obtain a certificate and work permit in exceptional schools. In Iran, education in exceptional education centers is daily and free. According to the published statistics by the Bureau of Exceptional Education in Tehran, Iran, 113 blind and 255 deaf children were studying in Tehran’s elementary schools. All of these children were selected to participate in the current study. To include mentally retarded children, ten exceptional education centers from five geographical areas in Tehran (North, South, East, West and Central) were selected. In each area, two centers (one for girls and one for boys) had been selected and then, the mentally retarded children in each center were included randomly by using probability proportional to size sampling. Totally, 250 mentally retarded children were included in this study. However, we excluded those with missing information. Overall, by considering blind and deaf children, 470 exceptional children including 62 blind, 182 deaf and 226 mentally retarded were included in the current study. Parents of children provided written informed consent to participate their children in the current study. The study was ethically approved by the Institute of Education Studies affiliated to Ministry of Education of Iran by this number: 900/51/38.

### Dietary habits

A pre-tested and validated dietary habit questionnaire was used to gather data on dietary intake [12–14]. Parents of children were asked to report the consumption frequency of their children for fruits, vegetables, milk, yogurt, cheese, red meat, poultry, fish and egg based on eleven multiple choice frequency response categories varying from “never” to 3 times/day. After data gathering, participants were categorized based on these response categories. Because of low number of participants, we combined some response categories to obtain three categories: <1time/ wk, 1–3 time/ wk and > 3 time/ wk. Presence of children in these categories was considered as the main exposure. To obtain total intake of dairy, we combined the intake of milk, yogurt and cheese. The frequency response categories for dairy were as follow: <1 time/day, 1–2 times/ day, > 2 times/ day. Difference in categories of dairy products compared with other food groups was due to high consumption of these products in children participated in the current study. In addition, intake of poultry and fish was combined to obtain intake of white meat. Also, white meat and red meat were combined to obtained intake of total meat. Dietary data were gathered using interview in this study.

Anthropometric measurements.

Height was measured by a fixed tape meter to the wall in the standard position without shoes while the shoulders, heels and buttocks were in contact with the wall with an accuracy of 1 cm. Weight was measured with minimal clothing and without shoes by analogue scale with a precision of 100 g. To determine stunting, we first calculated z-score for height-for-age. For this case, we compared the height for age index obtained in this study with the reference index reported by WHO 2007 for primary school students and adolescents using Anthro software [15]. The following formula was applied in this regard: Z-score = (observed value - median value of the reference population) / standard deviation value of reference population. In the current study, the height-for-age z-score of <-1 was considered as stunting [16].

### Statistical analysis

All statistical analyses were done by SPSS software (version 19.0; SPSS Inc, Chicago IL). To assess categorical variables across children with different disabilities, we used Chi-square test. We applied one-way ANOVA to compare continuous variables across children with different disabilities. To assess the association between dietary intakes and stunting, first, we categorized children based on frequency response categories for intake of fruits, vegetables, dairy, meat, red meat, white meat and egg. Binary logistic regression in crude and adjusted model was used to assess the association between intake of mentioned food groups and stunting. In adjusted model, age (continuous), sex (categorical) and other food groups (categorical) were adjusted. In these analyses, the first category of dietary intakes was considered as the reference category. To obtain the overall trend of odds ratios (OR) across increasing categories of dietary intakes, we considered these categories as an ordinal variable in the logistic regression models. In addition to total children, all analyses were separately done on mentally retarded children. P values less than 0.05 was considered as significant level.

## Results

Overall, 470 exceptional children including 226 mentally retarded, 182 deaf and 62 blind participated in the current study. Mean age of total children was 10.02 ± 2.04 years and 45.1% were female. The prevalence of stunting among children was 50.6%. In addition, stunting was prevalent among 71.7% of mentally retarded children, 36.3% of those who were deaf and 17.7% of blind children.

General characteristics and dietary intakes of exceptional children are shown in Table 1. Mentally retarded children had higher age, weight, were more likely to be female and stunted compared with children who were deaf or blind. In terms of dietary intakes, mentally retarded children had greater intake of vegetables, meat, red meat and white meat compared with other children.

**Table 1:** General characteristics and dietary intake of exceptional children (*n* = 470)

Variables	Mentally retarded	Deaf	Blind	P^**^
N	226	182	62	
Age (y)^a^	10.57 ± 2.08	9.44 ± 1.85	9.75 ± 1.93	0.001
Sex (boy) (%)	49.6	58.8	62.9	0.022
Weight (kg)	34.30 ± 12.83	30.74 ± 11.16	33.18 ± 12.92	0.018
Height (cm)	129.83 ± 14.63	131.49 ± 13.14	137.40 ± 11.94	0.001
Height for age (Z-score)	-1.81 ± 1.5	-0.57 ± 1.5	0.08 ± 1.21	< 0.001
BMI for age (Z-score)	0.74 ± 1.72	-0.01 ± 1.76	-0.37 ± 2.22	< 0.001
Stunting (%)	71.7	36.3	17.7	0.001
Dietary intake				
Fruit (> 3 times/wk) (%)	71.7	41.2	53.2	0.449
Vegetables (> 3 times/wk) (%)	15.5	4.9	1.6	0.001
Meat (> 3 times/wk) (%)	26.5	9.3	9.7	0.001
Red meat (> 3 times/wk) (%)	30.5	12.6	17.7	0.041
White meat (> 3 times/wk) (%)	10.2	6	1.6	0.017
Egg (> 3 times/wk) (%)	21.2	14.3	9.7	0.292
Dairy (> 2 times/d) (%)	34.5	23.1	21	0.418

^a^Data are Mean ± SD or percent

^**^Obtained using chi- square or ANOVA, where appropriate

Multivariable-adjusted odds ratios and 95% confidence intervals for stunting across categories of food groups intake are indicated in Table 2. Compared with children in the lowest category of egg consumption (< 1 time/ wk), those who consumed egg 1–3 time/ wk were less likely to be stunted (OR: 0.41, 95% CI: 0.24–0.71); such that after controlling for age, sex and other dietary intakes, children who ate egg 1–3 times/ wk had 64% less odds for having stunting compared with those who ate it < 1 time/ wk (OR: 0.36, 95% CI: 0.21–0.64). In terms of other dietary factors including intake of fruits, vegetables, meat, red meat and white meat, no significant association was found with stunting either before or after adjusting for confounders.

**Table 2:** Multivariable-adjusted odds ratios and 95% confidence intervals for stunting across categories of food groups intake in total children

Dietary intake		< 1 times/wk	1–3 times/wk	> 3 times/wk	P-trend
Fruits					
Crude		1	0.74 (0.34–1.58)	0.83 (0.42–1.65)	0.82
Adjusted^a^		1	0.51 (0.22–1.19)	0.66 (0.30–1.42)	0.528
Vegetables					
Crude		1	1.55 (1.00-2.40)	1.52 (0.79–2.93)	0.051
Adjusted		1	1.41 (0.87–2.28)	1.04 (0.50–2.14)	0.163
Meat					
Crude		1	1.22 (0.72–2.07)	1.73 (0.91–3.30)	0.106
Adjusted		1	1.17 (0.65–2.09)	1.68 (0.82–3.41)	0.117
Red meat					
Crude		1	1.11 (0.65–1.88)	1.44 (0.79–2.65)	0.247
Adjusted		1	0.99 (0.55–1.78)	1.34 (0.69–2.61)	0.396
White meat					
Crude		1	1.26 (0.80–1.98)	1.16 (0.54–2.51)	0.431
Adjusted		1	1.13 (0.68–1.88)	1.03 (0.44–2.42)	0.447
Egg					
Crude		1	0.41 (0.24–0.71)	0.73 (0.38–1.40)	0.307
Adjusted		1	0.36 (0.21–0.64)	0.69 (0.35–1.39)	0.424

Data are OR (95% CI)

^a^adjusted for age, sex and other dietary intakes

**Fig. 1:**
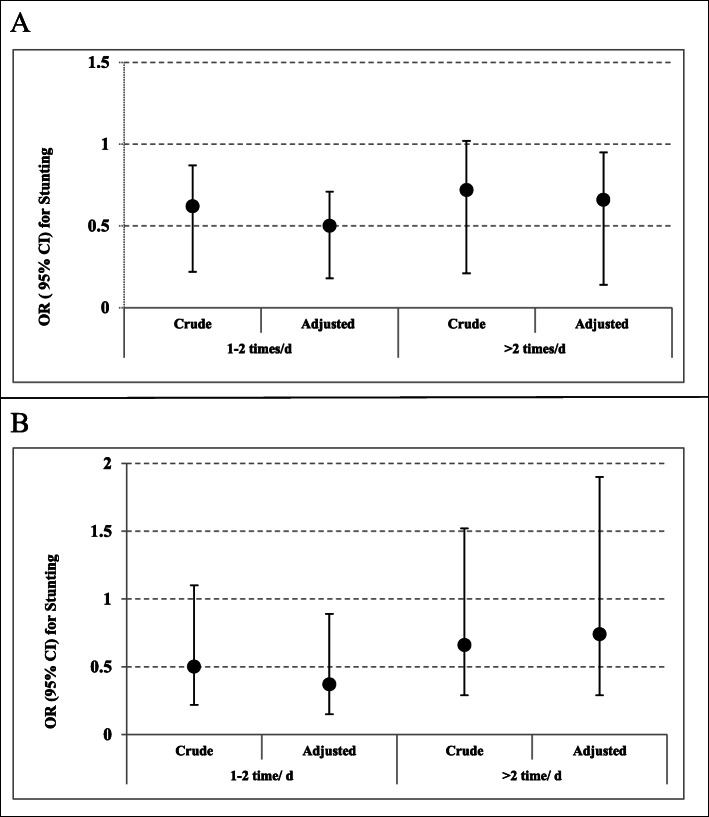
Multivariable-adjusted odds ratios and 95% confidence intervals for stunting across categories of dairy consumption in; **a** total children, **b** mentally retarded children. In adjusted model, age, sex and other dietary factors were controlled

Multivariable-adjusted odds ratios and 95% confidence intervals for stunting across categories of dairy consumption is presented in Fig. 1a. After controlling for age, sex and other dietary factors, children in the highest category of dairy consumption (> 2 times/day) were 50% less likely to be stunted compared with those in the lowest category (< 1 time/day) (OR: 0.50, 95% CI: 0.29–0.87).

Multivariable-adjusted odds ratios and 95% confidence intervals for stunting across categories of food groups intake in mentally retarded children are indicated in Table 3 and Fig. 1b. After taking potential confounders into account, mentally retarded children who consumed egg 1–3 times/wk had 62% lower odds of stunting compared with those who consumed < 1 time/wk (OR: 0.38, 95% CI: 0.16–0.89). No other significant association was seen between food groups and stunting among mentally retarded children in both crude and adjusted models.

**Table 3:** Multivariable-adjusted odds ratios and 95% confidence intervals for stunting across categories of food groups intake in mentally retarded children

Dietary intake		< 1 times/wk	1–3 times/wk	> 3 times/wk	P-trend
Fruits					
Crude		1	1.02 (0.22–1.10)	1.14 (0.41–3.15)	0.732
Adjusted model^a^		1	0.61 (0.15–2.41)	1.16 (0.34–3.97)	0.448
Vegetables					
Crude		1	1.08 (0.57–2.03)	1.00 (0.42–2.40)	0.926
Adjusted model		1	0.87 (0.41–1.85)	0.60 (0.21–1.73)	0.659
Meat					
Crude		1	1.78 (0.74–4.29)	1.58 (0.60–4.16)	0.553
Adjusted model		1	2.47 (0.66–9.28)	1.88 (0.23–15.18)	0.419
Red meat					
Crude		1	1.49 (0.66–3.36)	1.74 (0.71–4.28)	0.25
Adjusted model		1	0.92 (0.28–2.99)	1.60 (0.25–9.96)	0.351
White meat					
Crude		1	1.22 (0.58–2.57)	0.44 (0.15–1.25)	0.255
Adjusted model		1	1.09 (0.42–2.81)	0.45 (0.11–1.96)	0.733
Egg					
Crude		1	0.49 (0.23–1.06)	0.64 (0.25–1.61)	0.326
Adjusted model		1	0.38 (0.16–0.89)	0.61 (0.22–1.70)	0.395

Data are OR (95% CI)

^a^adjusted for age, sex and other dietary intakes

## Discussion

In the present study, we observed that more than half of studied children were stunted. Mentally retarded children were more affected compared with other children. Compared with children in the lowest category of dairy consumption, those in the highest category had lower odds of stunting. This association remained significant even after adjusting for covariates. In addition, moderate consumption of egg (1–3 time/wk) was inversely associated with stunting either before or after controlling for potential confounders. Such finding was also seen among mentally retarded children. No other significant association was seen between intakes of other food groups and stunting. Therefore, we conducted this first study to assess the association between intakes of food groups and stunting in exceptional children.

Stunting is related to high morbidity during childhood and it^’^s consequence in adulthood [17]. This condition can increase risk of overweight, obesity and related diseases such as metabolic syndrome in adulthood [17, 18]. Furthermore, stunting in children can affect the cognitive status and also reduce work capacity [5, 19]. Nutritional deficiency has an important role in etiology of stunting. A large number of studies have assessed the association between dietary intakes and stunting [5, 20, 21]. Also, the nutritional status of exceptional children has received less attention such as mentally retarded ones. Our findings indicated that a significant inverse association between dairy consumption and stunting. A prospective cohort study by Nguyen et al. were showed that consumption of dairy products was decreased risk of stunting [22]. In another cohort study, children who consumed higher amount of milk had better height growth, while consumption of other dairy products revealed no beneficial effect on linear growth [23]. However, the mentioned studies were conducted on healthy children, not exceptional ones. To our knowledge, we found no study assessing the association between dairy consumption and stunting in exceptional children.

Dairy products contain protein and calcium, which can explain the value of dairy products on linear growth in children [24, 25]. Evidence suggests adequate calcium intake is involved in bone mineralization and linear growth [26, 27]. Therefore, appropriate intake of calcium may decrease stunting phenomenon. Furthermore, dairy products are a rich source of high-biological value proteins that are required for linear growth [26, 28]. Consumption of these proteins stimulates the secretion insulin-like growth factor 1, known as contributing factor in linear growth of bone and mineralization [29]. On the other hand, children need more calcium and high-quality proteins (relative to weight) compared with adults because of rapid linear growth [30, 31]. Therefore, children who eat more dairy products, have higher bone and linear growth.

In current study, we also observed that moderate intake of egg was decreased risk of stunting among exceptional children and also those who were mentally retarded. Some studies had shown similar findings in healthy children. Results from a clinical trial showed that children who consumed 10 eggs per week during 6 months had better linear growth compared with those who ate ≤ 1 egg per week [32]. Similarly, Lee et al. reported [5] that consumption of egg in short stature children were significantly lower than those with normal height. In contrast, in a cross-sectional study on children in Ethiopia, Melaku et al. showed that higher adherence to a dietary pattern rich in egg was increased risk of stunting [33]. Perhaps this inconsistency in Melaku et al. [33] study was due to the evaluation of the egg-rich dietary pattern, not intake of egg alone, compared with present and previous studies. In addition, existence of contraversion findings can also be explained by the impact of different cooking methods in different communities.

In this study, we did not observe any association between the third tertile of egg consumption and stunting, which seems to be due to the small number of participants in this tertile and the widening of the confidence interval. Another reason might be cholesterol content of egg. Recently, stunted children were showed with high level of cholesterol compared to normal-height ones. Therefore, high intake of egg may stimulate stunting through elevation of total cholesterol concentrations.

Egg also, contains high- biological value proteins that are required for skeletal and linear growth. In addition, egg is known as a rich source of choline and essential fatty acids. Choline as a precursor of phospholipids is important for growth and development [34]. Some studies have confirmed that choline have beneficial effects on linear growth [35, 36]. In a study by Semba et al. [36] on rural Malawi children, stunted children had lower serum choline levels than those with normal height children [36]. In addition, cell proliferation that is the first step of linear growth needs proteins, choline and essential fatty acids that are available in egg [32, 35, 37]. Since in some communities these children are kept in day care institutions, there are restrictions on their food choices. Swallowing milk and eggs is easy and many children are interested in eating them. Therefore, the inclusion of milk and eggs, especially in snacks, can play an important role in the development of these children.

In the current study, we did not any association between other food groups intake including meat, fruits and vegetables and risk of stunting. This association remained non-significant even after adjustment for potential confounders. However, most previous studies showed an inverse association. For example, Lee et al. [5] observed that consumption of meat, fruits and vegetables in normal height children were higher than stunted children [5]. These food groups are rich in antioxidants, different types of vitamins and minerals and also proteins which all are necessary for growth and development [10]. Different physical condition of children participated in the current study compared with those who participated in the earlier studies might be a reason for the lack of significant association between these food groups (meat, fruits and vegetables) and stunting in the current study. Chewing meat, fruits and vegetables for exceptional children particularly those who were mentally retarded might be more difficult than healthy children. In addition, energy intake of exceptional children might be low due to their physical condition and it is possible that beneficial effects of meat, fruits and vegetables intakes on height might occur in the range of required energy intake. However, we did not measure energy intake in the current study. It is suggested that future studies consider this important variable for diet-disease relationships in exceptional children.

Unfortunately, this study suffered from some limitations which made our findings inconsistent. Based on the cross-sectional design of our study, we cannot confer a causal link between intakes of food groups and stunting. First, since the study design is cross-sectional, the cause-and-effect relationship between food groups and short stature is not clear. Therefore, these findings should be supported by prospective studies. Second, given that the use of questionnaires to collect nutritional data is a common method in studies, remembering the foods consumed can cause errors. However, this information was requested and recorded by an experienced and trained nutritionist. Although we adjusted some confounders for to assess the association between food groups intake and stunting, it seems that some other variables such as energy intake, parents’ height, economic status and physical activity need to be considered in future studies in which we did not collect data on these variables in the present study.

## Conclusions

In conclusion, prevalence of stunting among exceptional children participated in the current study was high (50.6%). In terms of contributing factors, intakes of dairy products and egg were inversely associated with risk of stunting. However, intakes of other food groups including meat, fruits and vegetables were not significantly related to stunting. According to the findings of this study, it is recommended to include milk and eggs in the diet of these children, especially in areas where these children are kept in institutions.

## Data Availability

Data will be available upon request from the corresponding author.

## Abbreviations

WHO: World health organization
OR: Odds ratios
